# The effectiveness of aesthetic care training on nurses’ perceptions of end-of-life care in patients with cancer: a quasi-experimental study

**DOI:** 10.1186/s12904-024-01343-4

**Published:** 2024-02-08

**Authors:** Sina Shahmohammadi, Parvin Mangolian shahrbabaki, Maryam Radmehr, Sedigheh Khodabandeh Shahraki

**Affiliations:** 1 Nursing Research Center, Kerman University of Medical Sciences, Kerman, Iran; 2 Community Health Research Center, Isfahan (Khorasgan) Branch, Islamic Azad University, Isfahan, Iran; 3 Reproductive health, Family and Population Research Center, Kerman University of Medical Sciences, Kerman, Iran

**Keywords:** Nurses, Aesthetic care training, End-of-life

## Abstract

**Background:**

Supportive end-of-life care plays a significant role for patients with cancer. Significantly, art and aesthetics in nursing are regarded as key components of nursing practice. They may contribute to supportive end-of-life care that nurses provide for patients with cancer. Therefore, this study aimed to examine the effectiveness of aesthetic care training on nurses’ perceptions of end-of-life care in patients with cancer.

**Methods:**

A quasi-experimental study was conducted with two groups of nurses working in the oncology wards of two hospitals in Kerman, Iran. A sample consisting of 100 nurses was selected by census and randomly assigned to an experimental group (*n* = 49) and a control group (*n* = 51). The experimental group received educational workshops on aesthetic care over four weekly-held 90-minute sessions. Both groups completed the Oncology Nurses’ Perceptions of End-Of-Life Care (ONPEoLC) Scale before, immediately after, and one month after the intervention. The data were analyzed with SPSS software version 21 using t-test, Chi-square, and repeated measures ANOVA. The significance level was set to *p* < 0.05.

**Results:**

The mean baseline scores on the ONPEoLC Scale were 163.08 ± 13.58 in the experimental group and 163.27 ± 14.57 in the control group. There was no statistically significant difference between the two groups (*P* > 0.05). Post-intervention mean scores in the experimental and control groups were 187.1 ± 18.22 and 159.11 ± 22.11, respectively, indicating a statistically significant difference between the two groups (*P* < 0.001). One month after the intervention, the experimental and control groups’ mean scores were 190.89 ± 11.13 and 165.80 ± 11.69, respectively, with a significant difference between the groups (*P* = 0.001).

**Conclusion:**

Based on the results of the present study, designing aesthetic care educational programs is an effective way to improve nurses’ understanding of end-of-life care. Therefore, it is recommended that nursing faculties and educational policymakers utilize aesthetic care training to improve the nurses’ perceptions of end-of-life care.

## Background

The provision of end-of-life (EoL) care is a complex issue that often presents challenges for patients, nurses, and other care providers who may not always feel adequately prepared to address its intricacies [1, 2]. EoL basically seeks to offer a “good and calm death” for patients and “peace and comfort” to patients’ families. The holistic care perspective of nurses in EoL care, which stems from their comprehension of caring for these patients, can assist them in delivering high-quality care that is grounded in value, support, and integrated with nursing care [3].

In this context, the implementation of treatment measures and nursing care is intensified, with nurses assuming a crucial and indispensable role in providing care for patients with cancer in the terminal stages of life [4]. Heidari et al. (2018) highlight a gap in the nursing education system regarding EoL care for patients with cancer [5]. There is concern that healthcare providers cannot provide the ground for the good passing of patients with cancer [6]. EoL care is based on attending to the care requirements of dying patients with cancer. This type of care aims to increase the patient’s sense of well-being, taking into account the patient’s needs, the nurse’s availability, the patient’s acceptance by nurses, and the willingness to compromise with the emotional actions of patients with cancer [7].

According to Abate et al. (2018), EoL care aims to alleviate or eliminate pain and other symptoms while simultaneously attending to a person’s psychological, social, spiritual, and physical needs. Therefore, nurses should be familiar with the concepts of EoL care through workshops, training, and formal or informal education in academic and hospital settings [8].

On the other hand, perception is a complex process that includes becoming aware of and comprehending sensory information, as well as adjusting and interpreting environmental impressions. Clearly, increasing nurses’ perceptions is an effective factor in enhancing EoL care for patients with cancer [9]. Lindvall et al. (2014) stated that insufficient knowledge and a misunderstanding of EoL care impede the implementation of palliative care and nurses’ attainment of clinical competence in patient care [10].

Aesthetics in nursing refers to the nurses’ perceptions of care in a unique and individualized manner, as well as their ability to provide care in situations they may encounter for the first time. As a result, aesthetic care can affect nurses’ perception of EoL care and is part of the EoL care quality [11].

Carper’s definition of aesthetics-based nursing care includes components such as kindness and compassion, clinical competence, creativity and curiosity, flexibility, valuing patients with cancer, empathy and sympathy with their experience, integration of nursing features, and the ability to plan holistic care with aesthetic talent [12, 13]. Numerous experts have concluded that knowledge of aesthetics is essential for nurses to achieve high levels of EoL care perception [14]. It is because EoL care is linked with certain characteristics to which nurses need to attend, including a proper understanding of creativity and purposefulness, skills, relationships, totalitarianism, personalization of care, application of empirical knowledge, and adherence to ethical principles [15].

According to Rees et al. (2020), EoL care education affects nurses’ knowledge of EoL care concepts, which may positively impact nursing practice [16]. The study by Oliveri et al. indicates that aesthetic treatments improve the quality of life of patients with cancer [17]. According to a study conducted in Iran by Radmehr et al. (2015), many caring behaviors, both for patients with cancer and nurses, are associated with an aesthetic experience [18]. In addition, research has revealed that the training and implementation of this type of care positively impact the quality of care behavior and the growth of the nursing profession, indicating the significance and necessity of implementing care based on aesthetics. Moreover, research shows that nurses are embracing the core values of nursing while continually striving to maintain the longstanding practice of caring for the dying. This includes preparing the family and the patient for this event and providing care during and after death [19]. The aesthetic dimension, according to Chinn and Kramer, refers to a profound perception of the significance of a given situation, which is inspired by an inner sense and transforms the experience into something that is not yet real but one that can be expressed through actions, behaviors, and attitudes [20]. Aesthetics-based nursing has been incorporated into nursing EoL care in a variety of ways, with empathy and compassion serving as the primary pillars across all cultures. Depending on their culture, nurses may express their emotions when confronted with the tactile experience of caring for certain patients with cancer through different art forms, such as music and drama [21].

According to Carper, the aesthetics of nursing care include an appreciation of and empathy for the experience of patients with cancer, the aggregation of the characteristics of nursing into a meaningful whole, and the capacity to design holistic care creatively. This aspect of nursing care encompasses the integration of various forms of nursing knowledge. The impact of this type of care is evident in EoL situations, where the components of holistic care with respect, growth with suffering, and coping with structural and cultural challenges are emphasized when facing patients dying of cancer [12, 13]. Similarly related are policies and regulations, which are necessary to advance nursing science and incorporate the aesthetic aspect of nursing into nursing education and practice. It is because nursing is committed to providing holistic human care, and the local cultural context and beliefs can influence nurses’ provision of the aesthetic aspect of nursing [21].

Previous studies have yielded conflicting findings on the impact of education on EoL care. In order to address this gap in the literature, the present study sought to examine the impact of aesthetic-based care training on nurses’ perceptions of EoL care for patients with cancer. The hypothesis posits that nurses who provide aesthetic care interventions exhibit greater perceptions of EoL care in patients with cancer.

## Methods

### Study type and setting

A quasi-experimental study was conducted from May to June 2022. The study was conducted in two hospitals, namely Afzalipour and Bahonar, located in the southeastern region of Iran. The participants were nurses working in the oncology departments of these hospitals. These hospitals are the largest teaching and medical centers in southeast Iran. They are also recognized as one of the leading institutions for providing advanced medical services and care for patients with cancer in the country.

### Sampling method

In this research, eligible nurses working in oncology departments in the southeast of the country (*n* = 107) were selected by the census method. Sampling was conducted from two distinct hospitals to mitigate the potential for interference and information exchange between the two groups of nurses. The two hospitals were labeled as either the experimental or control group through a lottery process. Afzalipour Hospital was considered the experimental group, while Bahanr Hospital was the control group.

**Fig. 1:**
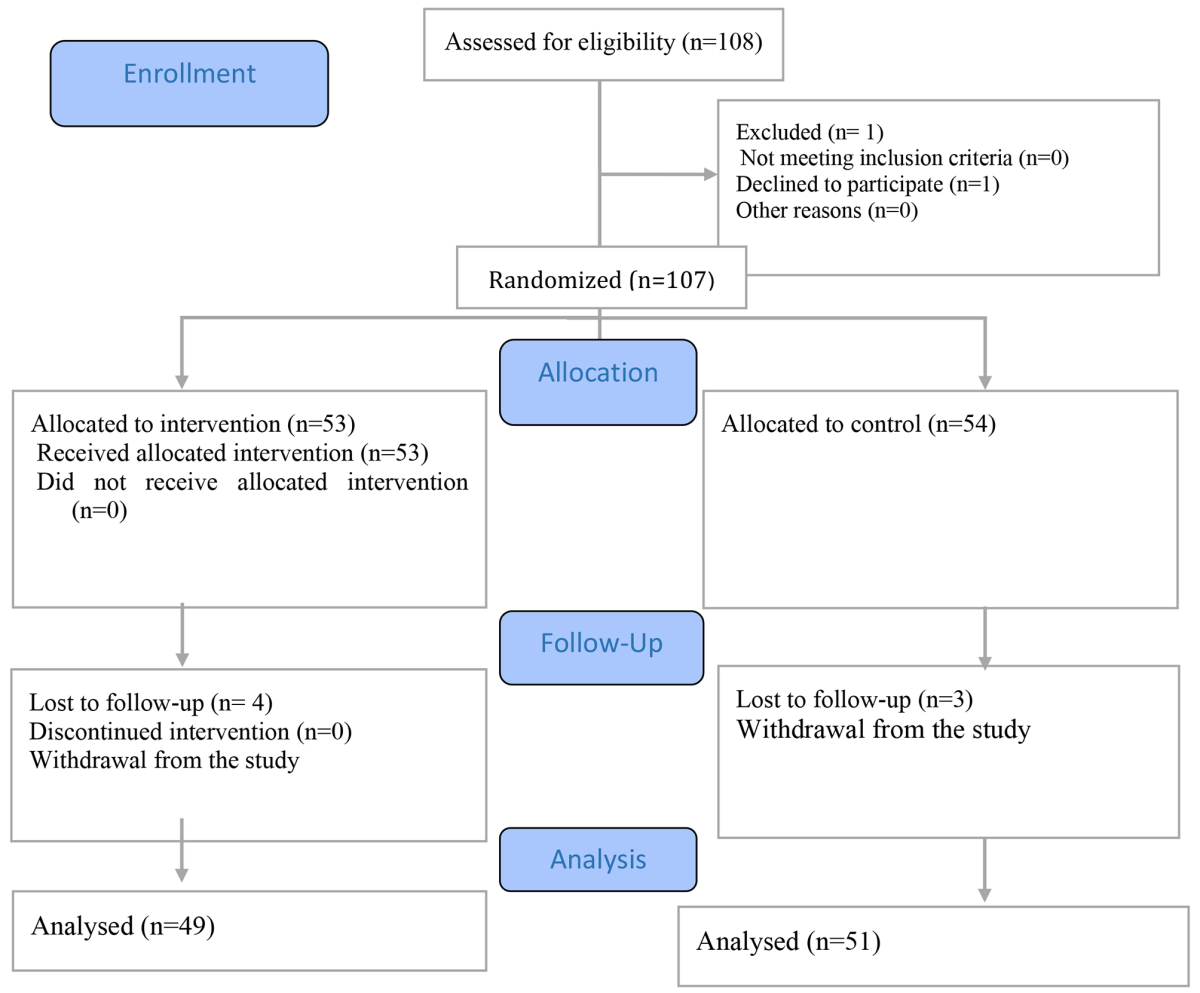
Study flow diagram

The inclusion criteria comprised a bachelor’s degree or higher in nursing and employment in an oncology department. The exclusion criteria included participants who missed at least three training sessions and failed to complete more than one-third of the questionnaire items. A total of 54 nurses served as the control group, while 53 participated in the experimental group. Four participants from the experimental group were excluded from the study. Two nurses were unable to participate in the training sessions due to contracting COVID-19, and two participants missed more than one-third of the sessions. Three nurses were excluded from the control group: two due to their unwillingness to continue participating and one nurse due to contracting COVID-19. The data from 100 participants were analyzed, with 49 in the experimental group and 51 in the control group (Fig. 1).

### Instrument

Two instruments were utilized for data collection:

1) Demographics form: The form covered demographic and occupational characteristics, such as age, gender, marital status, employment status, position, work tenure, work history in the oncology department, shift type, education, and history of aesthetic education and EoL care training.

2) Qaljeh (2016) developed the Oncology Nurses’ Perception of End-of-Life Care Scale (ONPEoLC). The scale comprises 42 statements that address four components: patient-centered care (statements 1–15), family-oriented care (statements 16–21), personal and professional characteristics of nurses (statements 22–29), and care in the context of structural and cultural challenges (statements 30–42). The items are scored based on a 5-point Likert scale, ranging from 1 (strongly disagree) to 5 (strongly agree). Scores may range from 42 to 210. A score below 98 signifies low perception, while a score between 98 and 154 indicates moderate perception and a score between 154 and 210 signifies high perception. Qaljeh et al. [22] developed the ONPEoLC scale after examining 310 nurses. The researchers confirmed the scale’s face validity qualitatively and determined its internal consistency by calculating Cronbach’s alpha coefficient, which yielded a value of 0.9. In the study, the reliability of the tool was assessed using the Alpha Cronbach technique, yielding a coefficient of 0.92.

### Data collection and intervention

There were three measurement time points in this study: pre-intervention, post-intervention, and follow-up.

### Before intervention

When the necessary permits were acquired, the hospital administration was reached out to collect the contact information of all eligible nurses (names, phone numbers, and e-mail addresses). The researchers contacted the nurses via telephone to pass on information regarding the research project. The study involved participants who volunteered to take part. The experimental group was assigned to Afzalipur Hospital nurses, while the control group consisted of nurses from Bahonar Hospital. Initially, the participants were presented with written consent forms by the researcher. The pretest Oncology Nurses’ perceptions of End-of-life Care (ONPEoLC) questionnaire was subsequently administered electronically to both groups. The questionnaire was designed using the Porsline software, and its link was sent via SMS to the participants. Upon accessing the link, participants were provided with a clear explanation of the questionnaire completion process, along with a guide to assist them in answering the questions. The questionnaire took nearly 20 min to complete. The post-test data was collected one month after the training program using the same questionnaire, i.e., the ONPEoLC scale.

### Intervention steps

The steps taken to implement the intervention were as follows:

The educational program schedule was coordinated with the experimental group and educational supervisor following the completion of the pretest.

The content of the educational intervention was compiled based on a review of the relevant literature and approved and finalized by the research team. The training content was approved by six experts, including four faculty members from the Department of Medical and Surgical Nursing at Kerman University of Medical Sciences and two experienced oncology nurses. This content was taken from clinical guidelines and nursing theory [14, 19, 23–27]. The content was presented in four 90-minute sessions held on a weekly basis. The experimental group received online training through a Sky room, which included lectures, Q&A sessions, discussions, PowerPoint slides, videos, and scenario presentations, all supervised by professors.

Throughout the entire training course, the nurses’ feedback was monitored through a discussion of their related experiences in the field of nursing care aesthetics, both from the perspective of patients and nurses. Their experiences allowed us to assess the course’s emotional training objectives.

The online class schedule was designed according to the nurses’ preferences. Each nurse was able to select a convenient time based on which lecturers presented the course. The course was also recorded, allowing nurses who were unable to attend the online class due to work commitments to access the training materials later on. The courses were conducted in group settings, with a minimum of five nurses participating in each group. An online group discussion was conducted at the conclusion of each session. They were instructed to withhold the educational materials from the control group. Both groups completed the ONPE**o**LC scale after the intervention and one month later as the follow-up. A description of the training program is provided in Table 1.

**Table 1:** The contents of the aesthetic care training sessions

	Objectives	Content of the session
Session 1	A basic understanding of and an introduction to the definitions provided for aesthetics	1 - Acquaintance with the experimental group members and communication of program goals and expectations 2- Conceptualization and definition of aesthetic care 3- Mechanisms by which aesthetic care influences the quality of EoL care 4- Free discussion
Session 2	Teaching the spiritual dimensions of nurses’ efforts to alleviate the suffering of patients with cancer through aesthetic nursing care	1- Describing the aesthetics of nursing care in a group discussion pertaining to the subjectivity of this concept and providing first-person accounts of this notion 2- Discussing the aesthetics of nursing care as an evident spirituality and citing narratives of patients with cancer and nurses’ lived experiences 3- Discussing and training aesthetic nursing care in terms of its impact on reducing helplessness sensed by patients with cancer as well as delightful care in reducing pain and suffering in patients with cancer 4- Citing narratives of patients with cancer and nurses’ lived experiences; requesting the nurses who attended the meeting to present some of their experiences in this field the following week; presenting video clips and scenarios
Session 3	Teaching the importance of a sense of unity with patients with cancer and the effect of nurses’ skills and expertise in providing aesthetic nursing care	1- Discussing a nurse’s empathy and companionship when providing aesthetic nursing care and presenting real-life examples of this care 2- Examining the effect of nurses’ competence and commitment in providing aesthetic care by citing narratives Assignment: Presenting their experiences and discussing each experience
Session 4	A discussion guided by the nurses on the aesthetics of nursing care and a summary of the contents	1- Discussion of the beauty of care and the nurses’ profound pleasure in alleviating the suffering of patients with cancer, which can motivate them to remain in their profession Assignment: Presenting their experiences and discussing each experience

In addition to being evaluated immediately following the intervention, the nurses were also monitored one month later. The participants filled out the scale again after one month. The educational content was provided to the control group when the intervention was completed.

### Statistical analysis

The data were analyzed with SPSS software version 21 using central tendency, dispersion, number, percent, independent sample t-test, paired sample t-test, Chi-square test, and repeated measures ANOVA. The significance level was set to *p* < 0.05.

### Ethical consideration

This article presents the findings of a master’s thesis (project No. 99,000,261) approved by the ethics committee affiliated with Kerman University of Medical Sciences (Ethics Code: IR.KMU.REC.1400.469). The objectives and procedure of the study were explained to the nurses, and they were assured that the information collected from them would be kept confidential and used solely for the purposes of the study. The participants were informed that they could withdraw at any time. At the conclusion of the study, two workshops and educational files were provided to the nurses in the control group, and the officials and participants were informed of the study’s findings.

## Results

The experimental group had a mean age of 30.08(± 5.65) years, and the control group had a mean age of 32.25(± 7.91) years. The majority of participants in both groups were female (88.2%), married (58.8%), and held a bachelor’s degree. In terms of demographic variables, there was no statistically significant difference between the two groups (*P* > 0.05) (Table 2).

**Table 2:** Comparison of the absolute and relative frequency distribution of demographic variables in control and experimental groups

Variable	Group	Experimental		Control		Test statistic	*P*-value	
		Frequency	Percent	Frequency	Percent			
Gender	Male	9	18.4	6	11.8	χ^2^ = 0.84	0.35	
	Female	40	81.6	45	88.2			
Marital status	Single	18	36.7	19	37.3	Fishers = 0.32	0.85	
	Married	30	61.2	30	58.8			
	Divorced	1	2	2	3.9			
Education	Bachelor’s	48	98	47	92.2	Fishers = 2.3	0.31	
	Master’s	1	2	2	3.9			
	Ph.D.	0	0	2	3.9			
Working shift	Fixed	5	10.2	5	9.8	χ^2^ = 0.004	0.94	
	Rotational	44	89.8	46	90.2			
Position	Head-nurse	4	8.2	3	5.9	χ^2^ = 0.34	0.84	
	Clinical nurse	43	87.8	45	88.2			
	Staff	2	4.1	3	5.9			
Attainment of information on end-of-life care	Yes	28	57.1	28	54.9	χ^2^ = 0.05	0.82	
	No	21	42.9	23	45.1			
History of caring for relatives	Yes	24	49	23	45.1	χ^2^ = 0.15	0.69	
	No	25	51	28	54.9			
Age (year)		Mean	Standard deviation	Mean	Standard deviation	Test statistic Independent t-test	*P*-value	
		30.08	5.65	32.25	7.91	t = -1.57	0.11	
Work experience		6.63	0.61	7.52	0.94	t = -1.54	0.11	
Work experience in oncology departments		2.6	0.54	3.21	0.6	t = -0.75	0.45	

As Table 3 indicates, the overall mean score of nurses’ perceptions of EoL care in the experimental group was moderate at baseline (163.08 ± 13.58). However, it steadily increased over time, as evidenced immediately (187.11 ± 8.22) and one month after the intervention (190.89 ± 11.13). In the experimental group, nurses’ knowledge of EoL care increased significantly over time (*P* = 0.001). In contrast, the mean score in the control group remained relatively stable and moderate from baseline (163.27 ± 14.57) to immediately after the intervention (159.11 ± 22.11) or one month after the intervention (165.8 ± 11.69). In the control group, the change in overall scores for nurses’ perceptions over time was not statistically significant (*P* = 0.13).

Also, based on the findings, the difference in the mean scores of the groups’ perceptions of EoL care was not significant at baseline (*P* = 0.94), while it was significant immediately and one month after the intervention (*P* = 0.001).

**Table 3:** Comparison of mean scores of nurses’ perceptions of end-of-life care at different time points in the experimental and control groups

Group Variable	Experimental	Control	Independent t-statistics	*P*-value
	Mean ± SD	Mean ± SD		
Before intervention	163.8 ± 13.58	163.27 ± 14.57	-0.06	0.94
Immediately after the intervention	187.1± 18.22	159.11 ± 22.11	6.89	0.001
One month after the intervention	190.89± 11.13	165.80 ± 11.69	10.98	0.001
Repeated measures analysis of variance test result	52.74	2.60		
*P*-value	0.001	0.13		

## Discussion

The study found that aesthetic care education had a significant effect on nurses’ perceptions of EoL care in patients with cancer. The experimental group differed significantly from the control group in this regard, which aligns with the findings of Mao et al. (2022) [27].

The authors argue that EoL nursing, viewed as an art, necessitates a holistic approach. This is because addressing the spiritual needs of patients with cancer at the EoL involves establishing trust, displaying respectful behavior, showing genuine love and affection, and respecting the dignity and religious beliefs of patients with cancer. These elements are all part of the aesthetic aspects of nursing care.

In fact, the spirituality described by patients with cancer and nurses at the EoL is exemplified by the nurses’ humane behavior and focus on the patient himself/herself rather than the patient’s illness. Even simple nursing duties can have high value if performed with respect for human dignity and health. Presenting and receiving these values requires an aesthetic approach that can be attained by educating nurses on EoL care [28].

Fesharaki and Radmehr (2020) confirmed that aesthetics-based nursing education is associated with nurses’ self-compassion traits. They observed that an enhancement in compassion traits corresponded to an improvement in aesthetic care quality, resulting in nurses being able to deliver better EoL care [29]. Another study demonstrated that nursing students’ perception of nursing care was enhanced through the implementation of self-compassion-based nursing education [29]. Rees et al.‘s study (2020) demonstrated the significance of nurses’ perceptions regarding appropriate EoL care for patients with cancer. They found that providing education on EoL care and utilizing interactive strategies can enhance nurses’ knowledge and adaptability to EoL, ultimately leading to positive effects on nursing practice [16].

According to the study’s findings, nurses experienced a sense of pleasure in their nursing care when they engaged in training sessions and exchanged experiences with their colleagues. This was attributed to an enhanced understanding and appreciation of EoL care. They stated that they were given a profound and lasting perception of care, which could lead to improved care quality. Multiple studies have found that providing training in EoL care and palliative care to nurses positively influences their attitudes and performance [30, 31]. Similarly, these trainings have been shown to enhance the performance of nursing students in areas such as communication, palliative care, symptom management, and the grieving process among patients with cancer [32]. The study’s findings suggest that aesthetic education may enhance compassion toward patients with cancer at their EoL. Alirezaee et al. (2021) demonstrated that compassion training aimed at enhancing the mental health of nursing students resulted in a significant increase in positive emotions and a significant decrease in negative emotions [33]. Lewis et al. (2016) found that caregivers who initially felt uncomfortable interacting with patients dying of cancer had a positive experience in providing EoL care after receiving training [34]. Nurses’ holistic perspective on EoL care, derived from their experience in caring for patients with cancer, enables them to deliver high-quality care that is grounded in values, support, and nursing care [29, 35].

Research suggests that nurses with high levels of compassion and aesthetics experience better mental health. This characteristic of nurses, combined with their strong belief in providing appropriate care, protects them from the stress associated with caregiving. Research suggests that nurses who possess elevated levels of compassion and aesthetics tend to experience improved mental health. Together with a firm conviction in giving proper care, this quality shields them from the emotional toll of their profession [36]. Hence, it is advisable to design and deliver aesthetic training that aligns with nurses’ specific cultural and contextual factors.

### Limitations of the study

As a result of the COVID-19 pandemic, cooperation among health service providers was limited; however, this was mitigated to a degree by measures such as the proper explanation of research objectives and, if necessary, the use of trained and trustable questioners. In addition, the pandemic caused fatigue and exacerbated the hectic working hours of nurses, which had an effect on their response rate; for this reason, training workshops and questionnaires were presented online. Given the scarcity of articles that directly addressed aesthetics in EoL care, and because aesthetics encompasses many different aspects, EoL studies were used in various aspects of aesthetics, such as compassion.

## Conclusions

The present research findings reveal that the aesthetic care training program has a desirable effect on increasing nurses’ perceptions of EoL care. Perceptions of care empower nurses to meet the needs and expectations of patients with cancer. Given the importance of care as the most important component of nursing practice, it is suggested that nursing managers improve the knowledge of nursing personnel in aesthetic care for patients with cancer in the final stages of life by holding in-service training courses. This is especially significant because aesthetic care training is a useful and low-cost method that can be implemented in all situations without needing special tools and causing side effects.

## Data Availability

The data are available upon request to the corresponding author after signing appropriate documents in line with ethical application and the decision of the Ethics Committee.

## Abbreviations

EoL: End of life
ONPEOLC: The Oncology Nurses’ Perceptions of End-of-Life Care
